# Ag_2_O Loading-Dependent Interfacial Properties of Ag_2_O/Co_3_O_4_ Composites for Gas-Phase and Electrochemical Sensing

**DOI:** 10.3390/molecules31203592

**Published:** 2026-10-09

**Authors:** Madiha Khan, Khouloud Abid, Viviana Bressi, Ahtisham Anjum, Claudia Espro, Giovanni Neri

**Affiliations:** 1 Department of Engineering, University of Messina, Contrada Di Dio, 1, 98166 Messina, Italy; 2 Department of Physics, Air University, Pakistan Air Force (PAF Complex), E-9, Islamabad 44000, Pakistan; 3 Institute of Advanced Technologies for Energy “Nicola Giordano”, 98126 Messina, Italy; 4 Research Department, Universidad Technologica ECOTEC, Km. 13.5 Samborondón, Samborondón EC092302, Ecuador; 5 Physics Department, King Fahd University of Petroleum & Minerals, Dhahran 31261, Saudi Arabia; 6 Interdisciplinary Research Center for Hydrogen Technologies and Carbon Management (IRC-HTCM), King Fahd University of Petroleum & Minerals, Dhahran 31261, Saudi Arabia

**Keywords:** Ag_2_O-loaded Co_3_O_4_, hydrogen conductometric gas sensor, screen-printed carbon electrode (SPCE), electrochemical uric acid sensor

## Abstract

Ag_2_O-loaded Co_3_O_4_ composites (Ag_2_O/Co_3_O_4_) were prepared by the sol–gel auto-combustion method. Their morphology, microstructure, and textural properties were characterized by scanning electron microscopy (SEM) coupled with energy-dispersive X-ray spectroscopy (EDX), Fourier transform infrared spectroscopy (FTIR), and Brunauer–Emmett–Teller (BET) analysis. The composites were evaluated as conductometric sensors for hydrogen (H_2_). The Ag_2_O/Co_3_O_4_−0.5 sensor showed an enhanced response to H_2_ over the investigated concentration range of 2000–10,000 ppm at an optimal operating temperature of 125 °C compared with pure Co_3_O_4_. The electrocatalytic activity of the composites was also investigated after spray-coating them onto screen-printed carbon electrodes (SPCEs). Cyclic voltammetry (CV) showed an enhanced response to uric acid for the Ag_2_O/Co_3_O_4_−1.0/SPCE compared with Co_3_O_4_/SPCE, with a sensitivity of 2.6 μA μM^−1^ cm^−2^, satisfactory reproducibility and stability, and a limit of detection (LoD) of 147 nM. These findings suggest that Ag_2_O/Co_3_O_4_ composites are promising materials for both gas and electrochemical sensing.

## 1. Introduction

Transition metal oxides (TMOs) are an important group of materials with a wide variety of structures, properties, and applications [1], including catalysis [2,3] and chemical sensing [4], in single-phase and/or mixed-phase forms. In general, semiconductor metal oxide gas sensors such as Co_3_O_4_, Fe_3_O_4_, CuO, SnO_2_, ZnO, WO_3_, ZrO_2,_ and others [2,5,6] have been extensively studied due to their varied electrical properties and reactivity to both oxidizing and reducing gases. To achieve the best gas sensitivity, the grain size must be less than twice the thickness of the depleting layer [7]. Therefore, metal oxide nanostructures have attracted significant attention for the development of chemical sensors or biosensors thanks to their unique morphologies, high surface area-to-volume ratios, and extraordinary physical and chemical properties [8].

In this regard, cobalt oxide (Co_3_O_4_) exhibits promising potential for applications in batteries, supercapacitors, solar cells, electrocatalysis, and sensing devices [1,5,7,9,10] because of its high specific surface area, good electrical properties, and electrochemical activity [7,11]. Co_3_O_4_ is a p-type semiconductor with excellent gas-sensing capabilities. In particular, the grain size of the particles in the gas-response layer affects both gas sensitivity and signal stability. Meanwhile, Co_3_O_4_ continues to attract considerable interest in electrocatalysis due to its unique electronic structure and excellent activity in oxygen evolution and reduction reactions, as well as in the electro-oxidation of carbohydrates, thiols, alcohols, and acetaminophen [1,6,12]. Researchers have prepared cobalt oxide (Co_3_O_4_) using several processes. Among these procedures are hydrothermal methods, microwave-assisted methods, co-precipitation, sonication, and sol–gel. The auto-combustion sol–gel approach has proven to be simple, repeatable, and low-cost, and is well-suited for large-scale applications [13]. However, it is only in the last one or two decades that significant interest, in both scientific and industrial domains, has developed as a result of the discovery of numerous benefits of alternative approaches.

Despite the above research efforts, single metal oxides have limited chemical, physical, and electrochemical properties. Recent interest in combining TMOs is increasing because their structures can accommodate several types of active metal cations, leading to a wide variety of mixed-oxide materials and thus yielding superior electrical, magnetic, and catalytic properties [14,15]. For example, vacuum-evaporated thin films of mixed cobalt and nickel oxide electrocatalysts were reported to exhibit high catalytic activity for oxygen reduction and evolution [16]. Enhanced activities of co-precipitated manganese-copper mixed oxides and manganese-iron or manganese-nickel mixed oxide catalysts, compared to those of their individual components, have been demonstrated [17]. Ag_2_O-loaded Co_3_O_4_ nanoparticles, Ag-based composites, and nanostructured Co_3_O_4_/CNT have been used as modifiers for electrochemical sensors, as reported in the literature [18]. The hydrothermal technique, a simple, efficient, and well-developed alternative approach for composite material preparation, has been used to obtain various metal oxides or mixed metal oxide composites [19].

Ag_2_O/Co_3_O_4_ composites have been investigated for gas and electrochemical sensing applications [20]. Regarding gas sensing, recent advancements in industrial processes have led to increased emissions and use of gases such as nitrogen dioxide (NO_2_), carbon monoxide (CO), and hydrogen (H_2_) [21,22]. In particular, H_2_ is increasingly used in industrial and energy applications, creating a growing need for rapid and reliable leak detection. Although H_2_ is non-toxic, its high flammability and potential accumulation in enclosed spaces pose significant safety risks, making continuous monitoring essential. Therefore, selective and fast-response sensors are required for H_2_ detection, particularly in confined environments and industrial settings where leakage and accumulation may occur. Metal-oxide-based sensors represent a promising approach for reliable H_2_ detection.

On the other hand, semiconductor metal oxides have shown great potential for electrochemical sensing applications toward biomolecule detection [23]. Uric acid (UA) is widely distributed in blood, beer, and foods [24]. Excessive uptake of UA is harmful, and it is therefore an analyte of interest in the food industry and clinical diagnostics. Inspired by the catalytic properties of Ag_2_O and Co_3_O_4_, as well as the possible synergistic effects of binary composites, we demonstrated in this work the feasibility of preparing a silver oxide (Ag_2_O)-loaded cobalt oxide composite also as an active electrochemical sensor for this biomolecule [25].

The synthesis of Ag_2_O on the surface of Co_3_O_4_ particles was performed via a simple chemical pathway. A comprehensive examination of the material characteristics in terms of morphology by scanning electron microscopy (SEM), chemical structure by FTIR, porosity, and Brunauer–Emmett–Teller (BET) surface area was carried out. The sensing material’s properties toward the hydrogen target gas were thoroughly investigated with respect to sensitivity, response time, and conductivity. The sensing process is also examined, along with possible explanations for the improved sensing performance of Ag_2_O-loaded Co_3_O_4_ composites. The results show that the developed sensors are easy to fabricate and exhibit a pronounced response toward H_2_ at moderate operating temperatures. Ag_2_O/Co_3_O_4_ composite has also emerged as a promising electrochemical sensing material for sensor modification for selective uric acid detection owing to its favorable electrocatalytic activity and charge-transfer properties. Electrochemical characterization by cyclic voltammetry and electrochemical impedance spectroscopy has demonstrated its suitability as a sensing platform, while its analytical performance highlights its potential for selective, stable, and reliable electrochemical sensing of biomolecules.

## 2. Results and Discussions

### 2.1. Characterization of Ag_2_O/Co_3_O_4_ Composites

**Figure 1:**
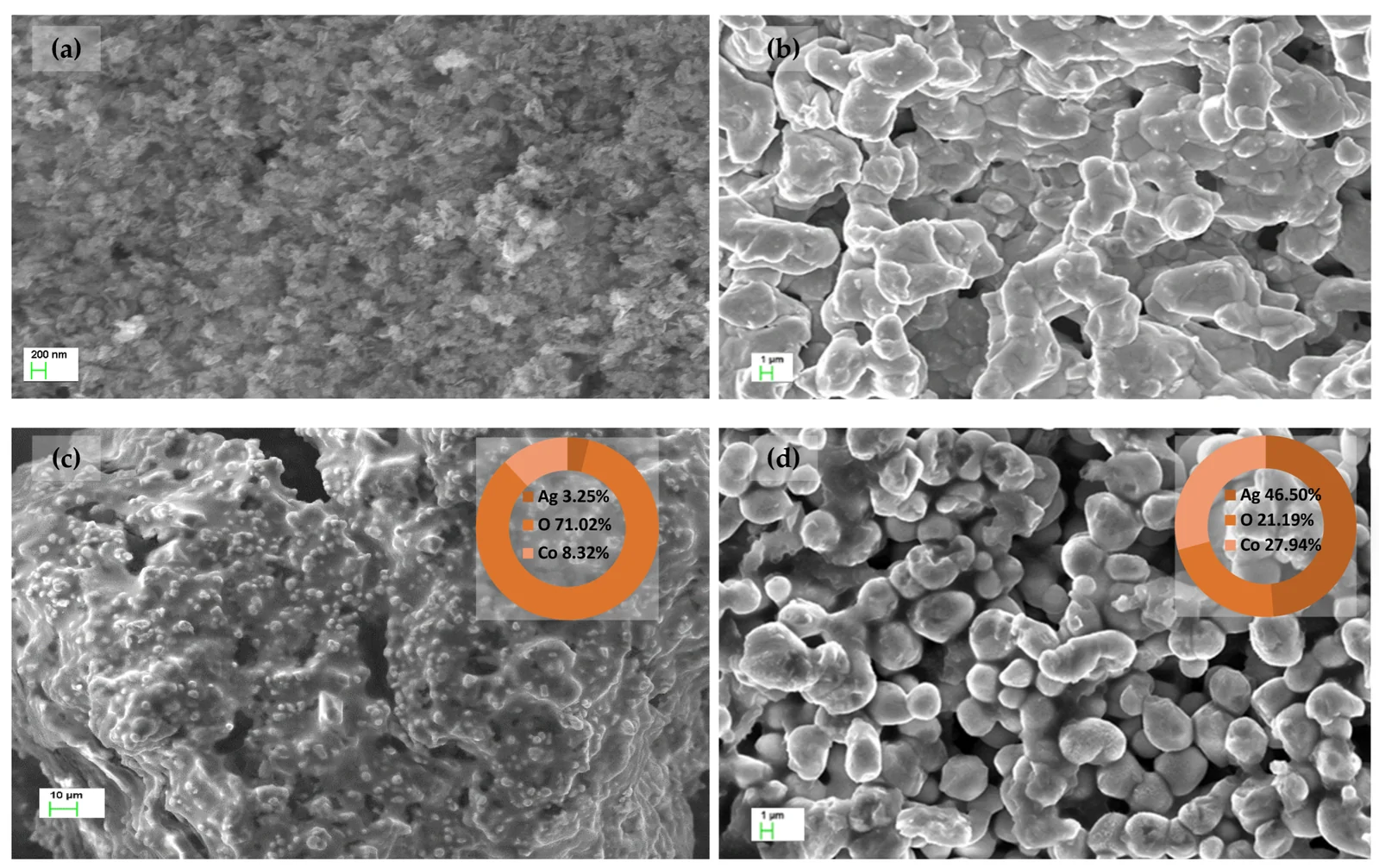
SEM images of (**a**) pure Co_3_O_4_, (**b**) pure Ag_2_O, (**c**) Ag_2_O/Co_3_O_4_−0.5, and (**d**) Ag_2_O/Co_3_O_4_−1.0 composites, with the corresponding EDX elemental compositions.

The morphology and microstructure of the Ag_2_O/Co_3_O_4_ composites were investigated by scanning electron microscopy (SEM) coupled with energy-dispersive X-ray spectroscopy (EDX), as shown in Figure 1. The SEM images of pristine Co_3_O_4_ reveal a relatively uniform, fine-grained morphology characterized by densely packed particles forming agglomerated clusters. In contrast, pure Ag_2_O exhibits a more irregular structure, composed of interconnected particles with non-uniform shapes. Upon formation of the Ag_2_O/Co_3_O_4_ composites, a distinct morphological evolution is observed. The composite samples display a heterogeneous structure consisting of Co_3_O_4_ microparticles decorated with smaller Ag_2_O particles, leading to a rougher and more porous surface. This morphological evolution suggests successful integration of the Ag-containing component within the Co_3_O_4_-based material, potentially contributing to the observed catalytic and sensing behavior. The EDX analysis (insets in Figure 1c,d) confirms the presence of Co, Ag, and O, with no detectable impurities. The corresponding elemental maps indicate a homogeneous distribution of Ag within the Co_3_O_4_-based material, confirming the successful incorporation of the Ag-containing component. It should be noted that EDX provides elemental information and does not independently determine the oxidation state of Ag.

A summary of the textural properties of the investigated samples, including specific surface area, pore volume, and pore radius, as determined by Brunauer–Emmett–Teller (BET) analysis from N_2_ adsorption–desorption isotherms, is reported in Table 1. Compared to the pristine oxides, the Ag_2_O/Co_3_O_4_ composites exhibit a significant decrease in specific surface area. In particular, the surface area decreases to 30.8 m^2^ g^−1^ for the Ag_2_O/Co_3_O_4_−0.5sample. Interestingly, a partial recovery of surface area is observed for the Ag_2_O/Co_3_O_4_−1.0 composite, reaching 38.4 m^2^ g^−1^. As reported in the literature, the initial decrease could be due to partial obstruction of the pores by Ag_2_O crystallites, confirming the interaction between the two materials [26]. The subsequent increase at higher Ag_2_O loading may be associated with a more homogeneous dispersion of Ag_2_O and the formation of a less compact structure, which partially restores the accessible surface area. A similar trend is observed for the pore volume, which decreases significantly in the composites compared to the pure oxides, further supporting the pore-blocking effect. In contrast, the pore radius shows only minor variations, indicating that the overall pore structure is largely preserved despite the incorporation of Ag_2_O.

**Figure 2:**
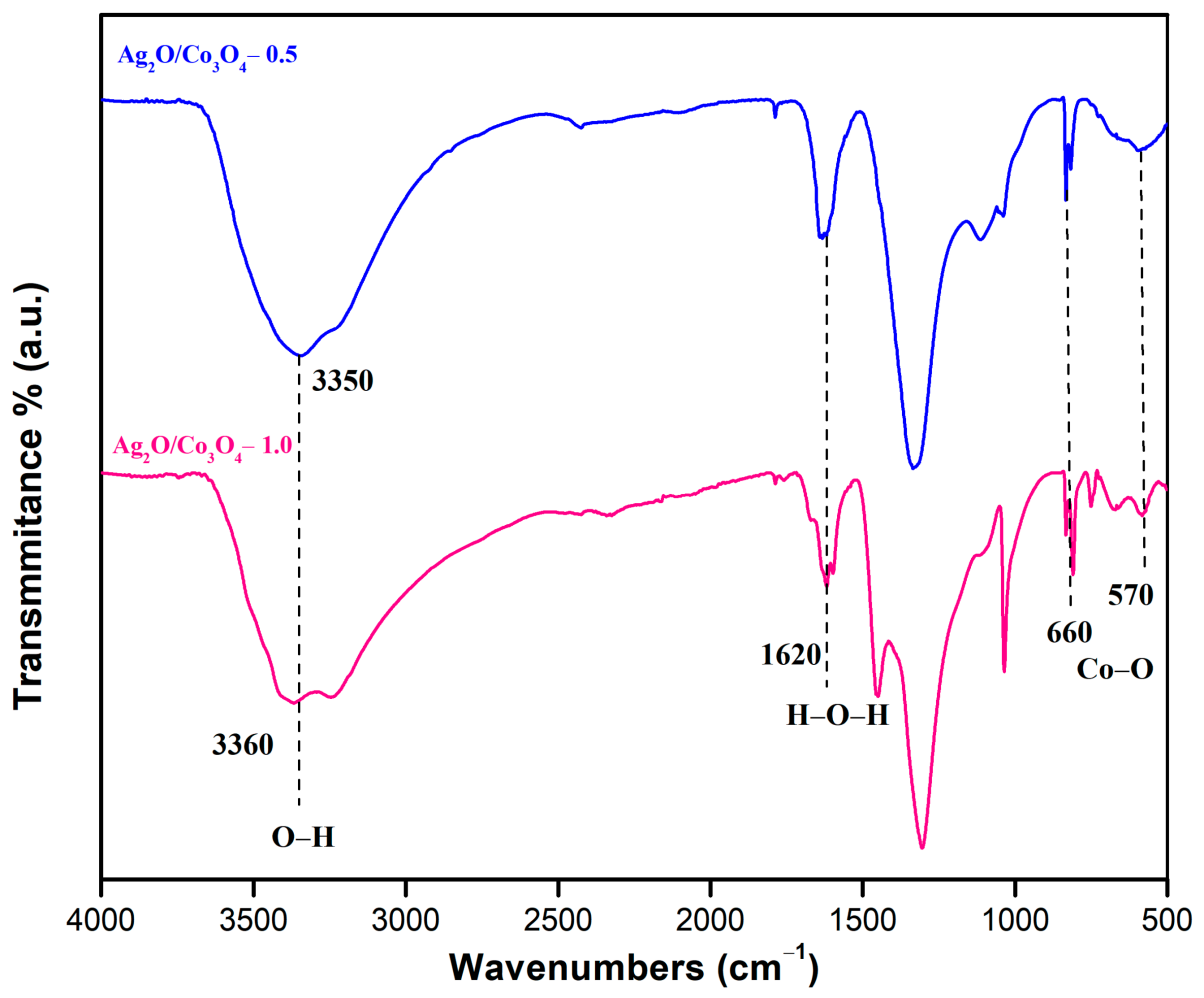
FTIR spectra of Ag_2_O/Co_3_O_4_−0.5 (blue line) and Ag_2_O/Co_3_O_4_−1.0 (pink line).

The FTIR spectra of Ag_2_O/Co_3_O_4_ composites (Figure 2) show the typical features of Co_3_O_4_, confirming that the spinel structure is preserved after Ag_2_O incorporation. A broad band at ~3200–3500 cm^−1^ is attributed to O–H stretching of adsorbed water [27], while the signal at ~1600 cm^−1^ corresponds to its bending mode. The characteristic Co–O vibrations appear at ~660 and ~570 cm^−1^ [28], associated with tetrahedral and octahedral sites of Co_3_O_4_. The low-wavenumber region deserves particular consideration in the Ag_2_O/Co_3_O_4_ composites. Ag–O stretching vibrations are commonly reported around 600 cm^−1^, which falls within the spectral region dominated by the characteristic Co–O lattice vibrations of Co_3_O_4_ observed at approximately 570 and 660 cm^−1^. Considering the very low nominal Ag_2_O loading (0.5–1.0 wt%) relative to the Co_3_O_4_ matrix, the Ag–O contribution is expected to be comparatively weak and may overlap with the much more intense Co–O absorptions, preventing its unambiguous resolution as an independent FTIR band. Therefore, the absence of a distinct band at approximately 600 cm^−1^ should not, by itself, be interpreted as evidence for the absence of Ag_2_O in the composite.

### 2.2. Gas Sensor for Hydrogen Detection

**Figure 3:**
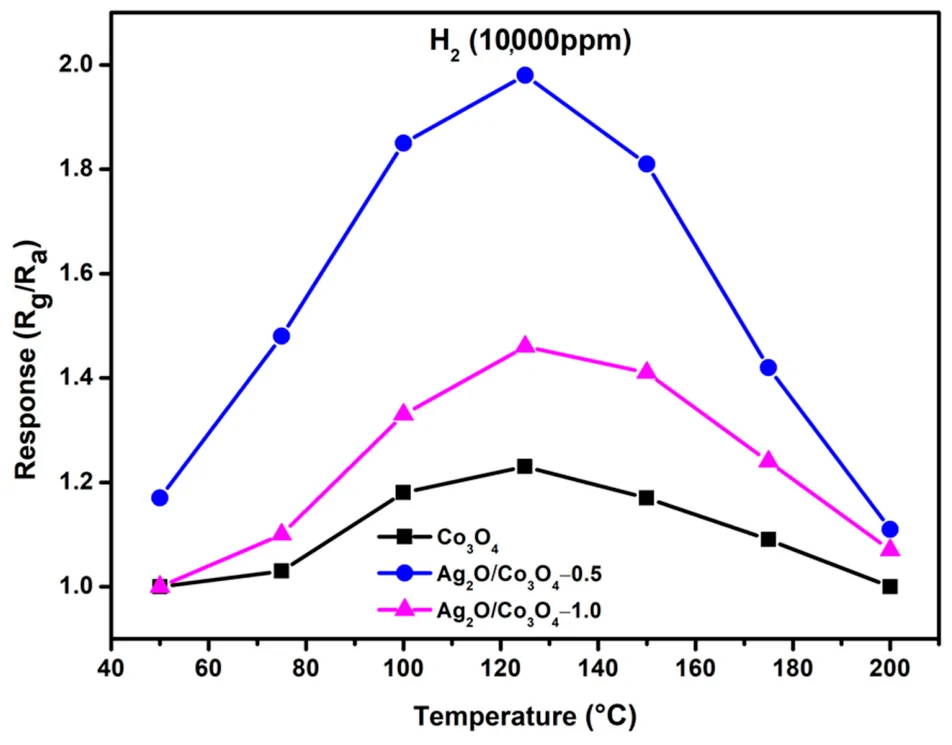
Response of the H_2_ gas sensors as a function of operating temperature (50–200 °C) at a fixed H_2_ concentration of 10,000 ppm for Co_3_O_4_, Ag_2_O/Co_3_O_4_−0.5, and Ag_2_O/Co_3_O_4_−1.0.

To evaluate the performance of the hydrogen sensors, the operating temperature was varied to determine the optimal temperature for each sensor. Figure 3 shows the temperature dependence of the responses of the three sensors over the range of 50–200 °C at a fixed H_2_ concentration of 10,000 ppm. The response of the Ag_2_O-loaded Co_3_O_4_ composite sensor (Ag_2_O/Co_3_O_4_−0.5) was greater than that of the pristine Co_3_O_4_ sensor at all temperatures.

The response of all sensors increased with increasing operating temperature up to 125 °C and then decreased. At an H_2_ concentration of 10,000 ppm, the sensor response is determined by a balance between hydrogen adsorption and desorption rates and the surface reactivity of adsorbed hydrogen with adsorbed oxygen species. To some extent, increasing the operating temperature promotes hydrogen adsorption, thereby improving the reaction rate at the sensor surface and enhancing the gas response. At higher temperatures, the gas response decreases owing to hydrogen desorption, which reduces the amount of hydrogen adsorbed on the sensor surface. Therefore, all subsequent gas-sensing experiments were conducted at 125 °C, the identified optimal operating temperature.

**Figure 4:**
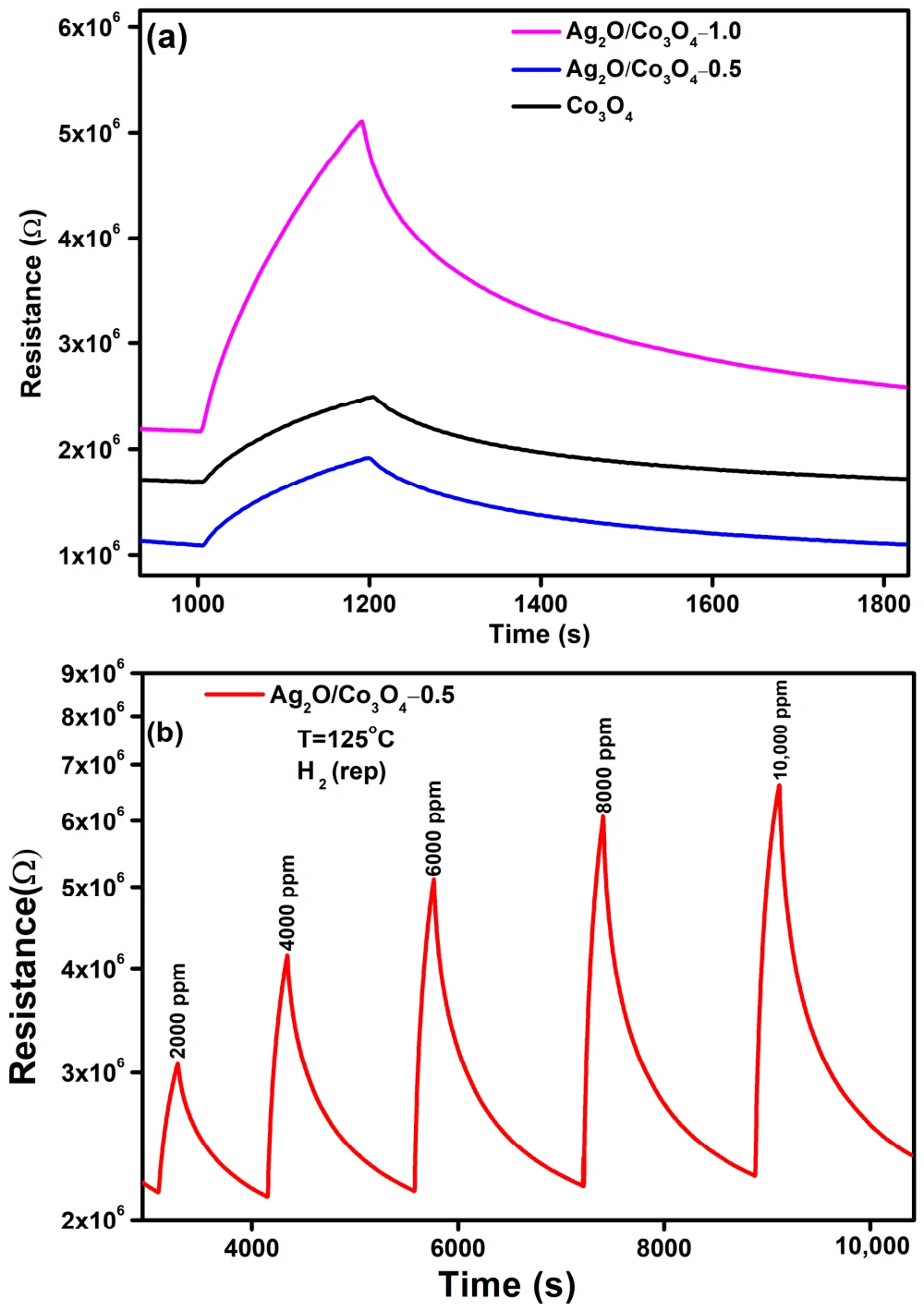
(**a**) Dynamic resistance response of Co_3_O_4_, Ag_2_O/Co_3_O_4_−0.5, and Ag_2_O/Co_3_O_4_−0.5 sensors upon H_2_ exposure at 125 °C; (**b**) dynamic response and recovery behavior of the Ag_2_O/Co_3_O_4_−0.5 sensor upon exposure to increasing H_2_ concentrations from 2000 to 10,000 ppm at 125 °C.

All sensors showed increased resistance upon exposure to hydrogen gas, indicating that the addition of Ag_2_O to Co_3_O_4_ did not alter the p-type semiconducting properties of pure Co_3_O_4_. Figure 4a shows the response of the three sensors to hydrogen over a concentration range of 2000–10,000 ppm. The Ag_2_O/Co_3_O_4_−0.5 sensor showed the best response and recovery behavior, as shown in Figure 4b.

**Figure 5:**
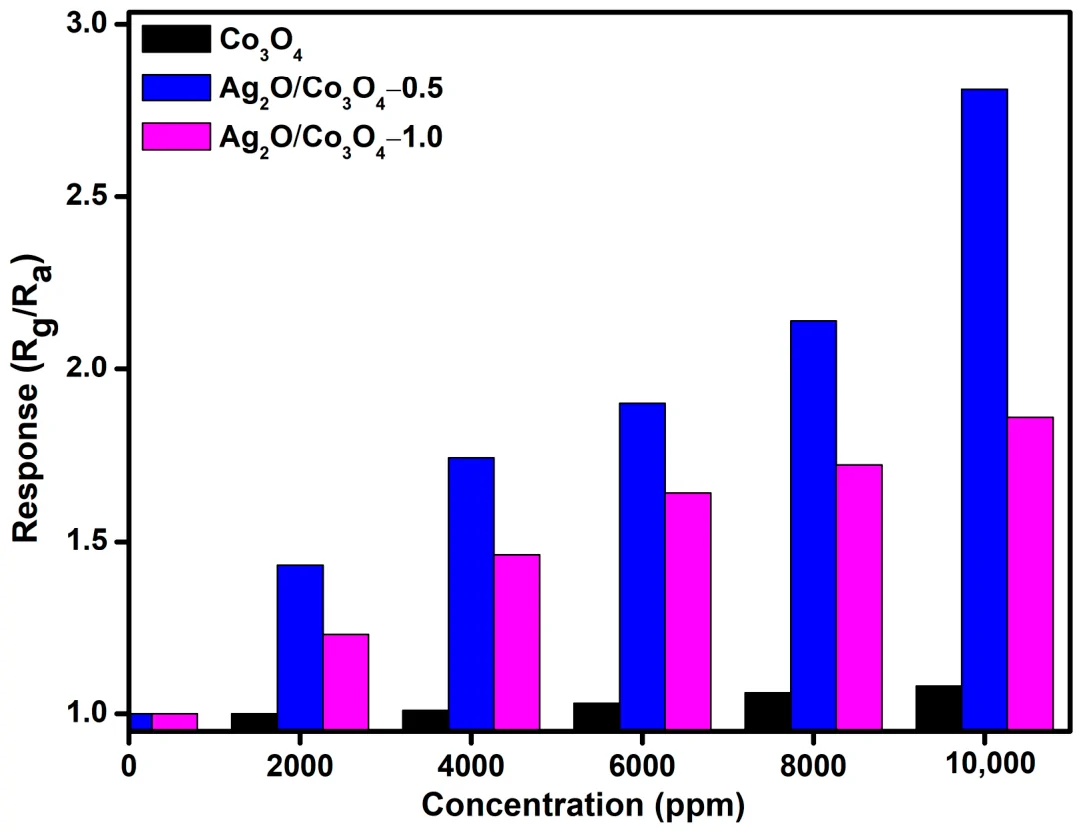
Sensor response toward H_2_ as a function of gas concentration at 125 °C for Co_3_O_4_, Ag_2_O/Co_3_O_4_−0.5, and Ag_2_O/Co_3_O_4_−1.0 sensors.

The responses of all sensors at 125 °C are shown in Figure 5. The Ag_2_O loaded Co_3_O_4_ composite sensor showed a higher response to H_2_ than the pristine Co_3_O_4_ sensor over the entire investigated concentration range. The difference in response among the three sensors was minimal at low H_2_ concentrations, whereas at higher concentrations, the Ag_2_O/Co_3_O_4_−0.5 sensor exhibited a significantly higher response than the pristine Co_3_O_4_ and Ag_2_O/Co_3_O_4_−1.0 sensors.

The sensor response was calculated according to Equation (1). After each H_2_ pulse, the signal returned to its initial baseline value, indicating that the sensing process was reversible under the investigated conditions. Furthermore, at 10,000 ppm H_2_, the Ag_2_O/Co_3_O_4_−0.5 sensor exhibited a response (R_(g)_/R_(a)_) of approximately 2.8.

**Figure 6:**
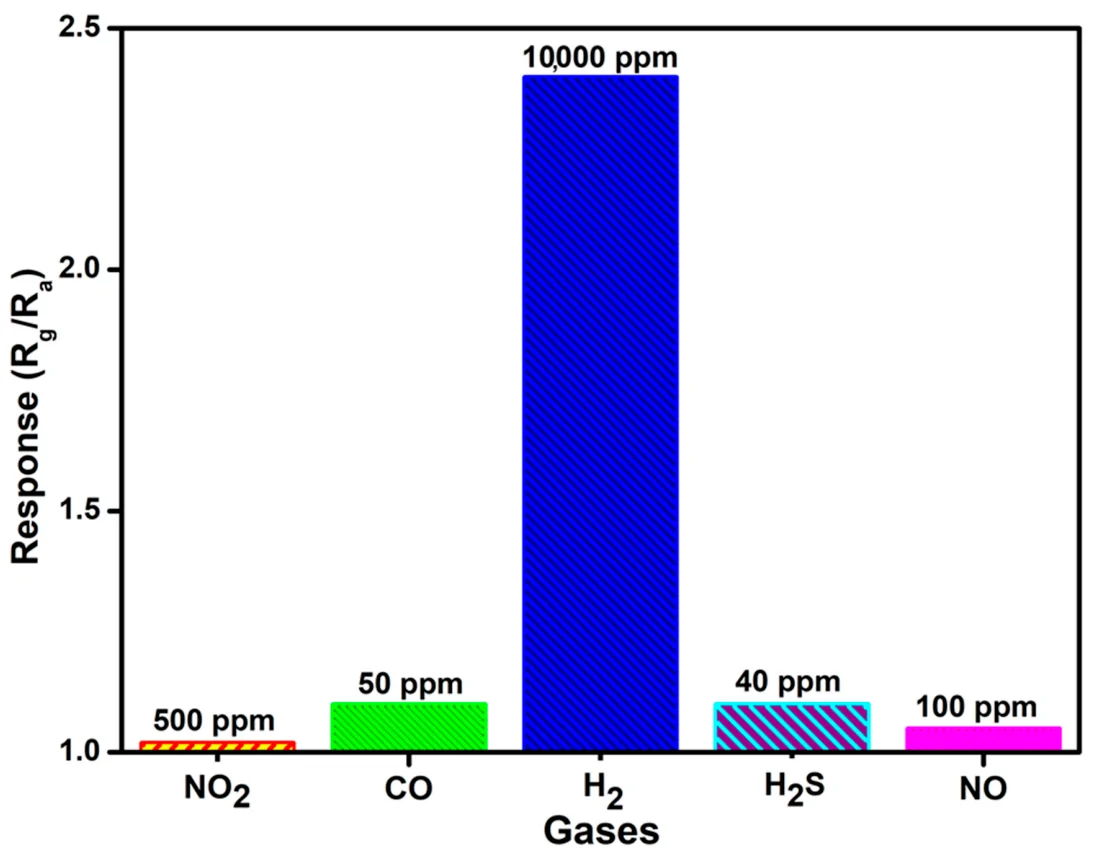
Response of the fabricated sensors for H_2,_ NO_2_, CO, H_2_S, and NO gases at their respective gas concentrations (ppm, as indicated in the figure), 125 °C, with Ag_2_O/Co_3_O_4_−0.5.

The response of the Ag_2_O/Co_3_O_4_−0.5 sensor toward potentially interfering gases was also investigated (Figure 6). NO, CO, NO_2_, and H_2_S were selected as representative potentially interfering gases. Since H_2_ and the interfering gases were tested at different concentrations (as reported in Figure 6), these measurements should be regarded as a preliminary interference screening under the investigated conditions rather than as a quantitative assessment of chemical selectivity. Direct comparison of selectivity would require measurements at equivalent or otherwise appropriately normalized gas concentrations.

The change in resistance upon H_2_ exposure can be explained considering the p-type semiconducting behavior of Co_3_O_4_. In air, oxygen molecules adsorb onto the Co_3_O_4_ surface and capture electrons from the semiconductor, forming ionized oxygen species (O_2_^−^, O^−^, and O^2−^, depending on the operating temperature, specific surface area, and particle size) [29,30]. The adsorption and ionization of oxygen species can be described by the following reactions:

(1) $$O_{2}\left(gas\right)\to O_{2}\left(adsorbed\right)$$

(2) $$O_{2}\left(gas\right)+e^{-}\to \;{O_{2}}^{-}\left(adsorbed\right)$$

(3) $$O_{2}^{-}\left(adsorbed\right)+e^{-}\;{↔\;2O}^{-}\left(adsorbed\right)$$

(4) $$O^{-}\left(adsorbed\right)+e^{-}\to O^{2-}\left(adsorbed\right)$$

The withdrawal of electrons from p-type Co_3_O_4_ increases the hole concentration in the near-surface region, resulting in a relatively low electrical resistance. Under a constant air atmosphere, the equilibrium between oxygen adsorption and ionization stabilizes the baseline resistance of the sensing layer. Upon exposure to H_2_, the reducing gas reacts with the chemisorbed oxygen species, producing H_2_O and releasing electrons back to the Co_3_O_4_ surface. For example, considering O^−^ as the reactive adsorbed oxygen species, the reaction can be represented as follows [29,30]:

(5) $$H_{2}\left(gas\right){+\;O}^{-}\left(adsorbed\right){\to H}_{2}O+\;e^{-}$$

**Scheme 1:**
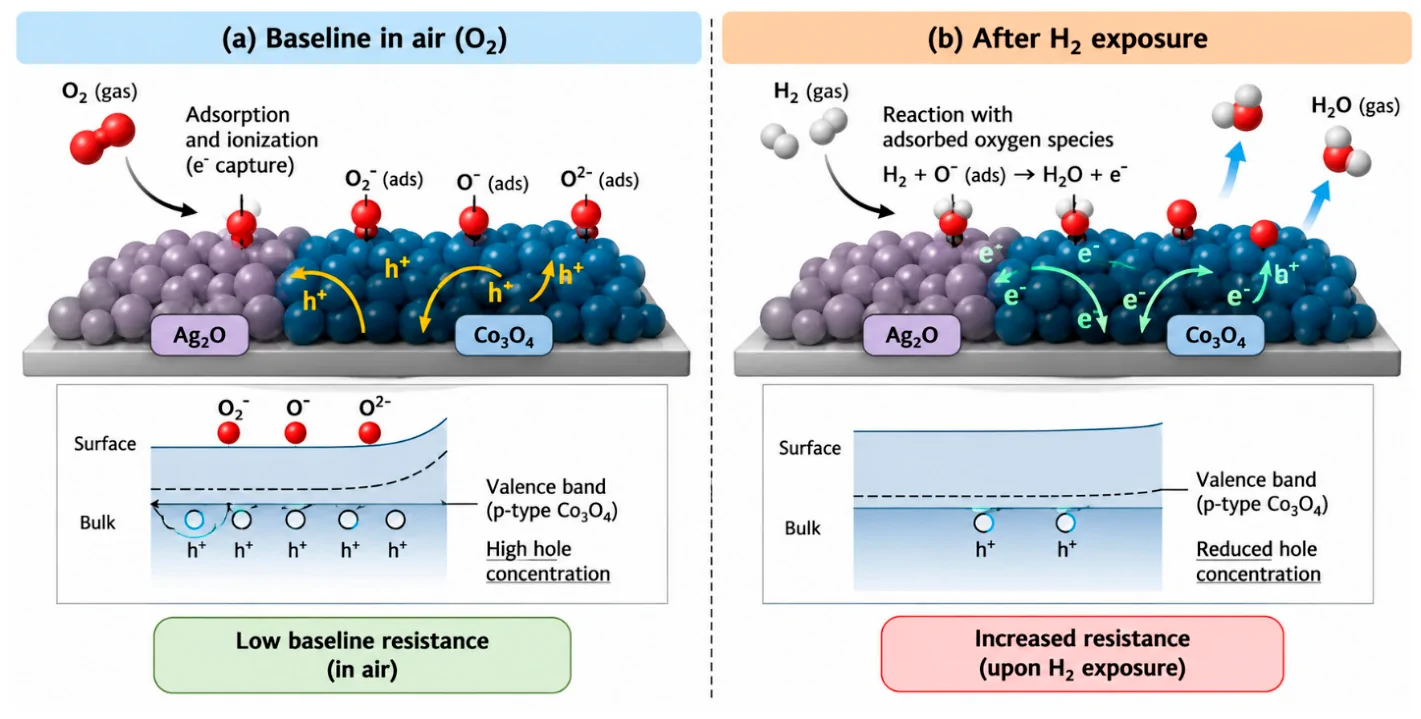
Schematic representation of the proposed H_2_ sensing mechanism at the Ag_2_O/Co_3_O_4_ surface (**a**) before and (**b**) after H_2_ exposure.

The electrons released during this surface reaction recombine with holes, decreasing the majority-carrier concentration in p-type Co_3_O_4_. Consequently, the hole concentration decreases, leading to an increase in the electrical resistance of the p-type Co_3_O_4_ sensing layer. When H_2_ is removed and the sensor is re-exposed to air, oxygen is readsorbed onto the surface, electrons are again withdrawn from the semiconductor, and the surface hole concentration is restored. As a result, the resistance progressively returns to its initial value. This reversible surface process is consistent with the experimentally observed increase in resistance upon H_2_ exposure and subsequent baseline recovery. The incorporation of Ag_2_O can further influence this surface-mediated sensing process by modifying the interfacial charge distribution and providing additional active sites for oxygen adsorption and H_2_ interaction. Consequently, the Ag_2_O/Co_3_O_4_ heterointerface can enhance the modulation of the charge-carrier concentration upon exposure to H_2_, resulting in a larger resistance variation than that observed for pristine Co_3_O_4_. This proposed surface sensing mechanism before and after H_2_ exposure is schematically illustrated in Scheme 1.

A comparison of the general and gas-sensing characteristics (preparation method, operating temperature, gas detected, etc.) of some Co_3_O_4_ and Ag_2_O-based sensors reported in the literature with our sensor described in the present work is shown in Table 2.

### 2.3. Electrochemical Uric Acid Sensor

**Figure 7:**
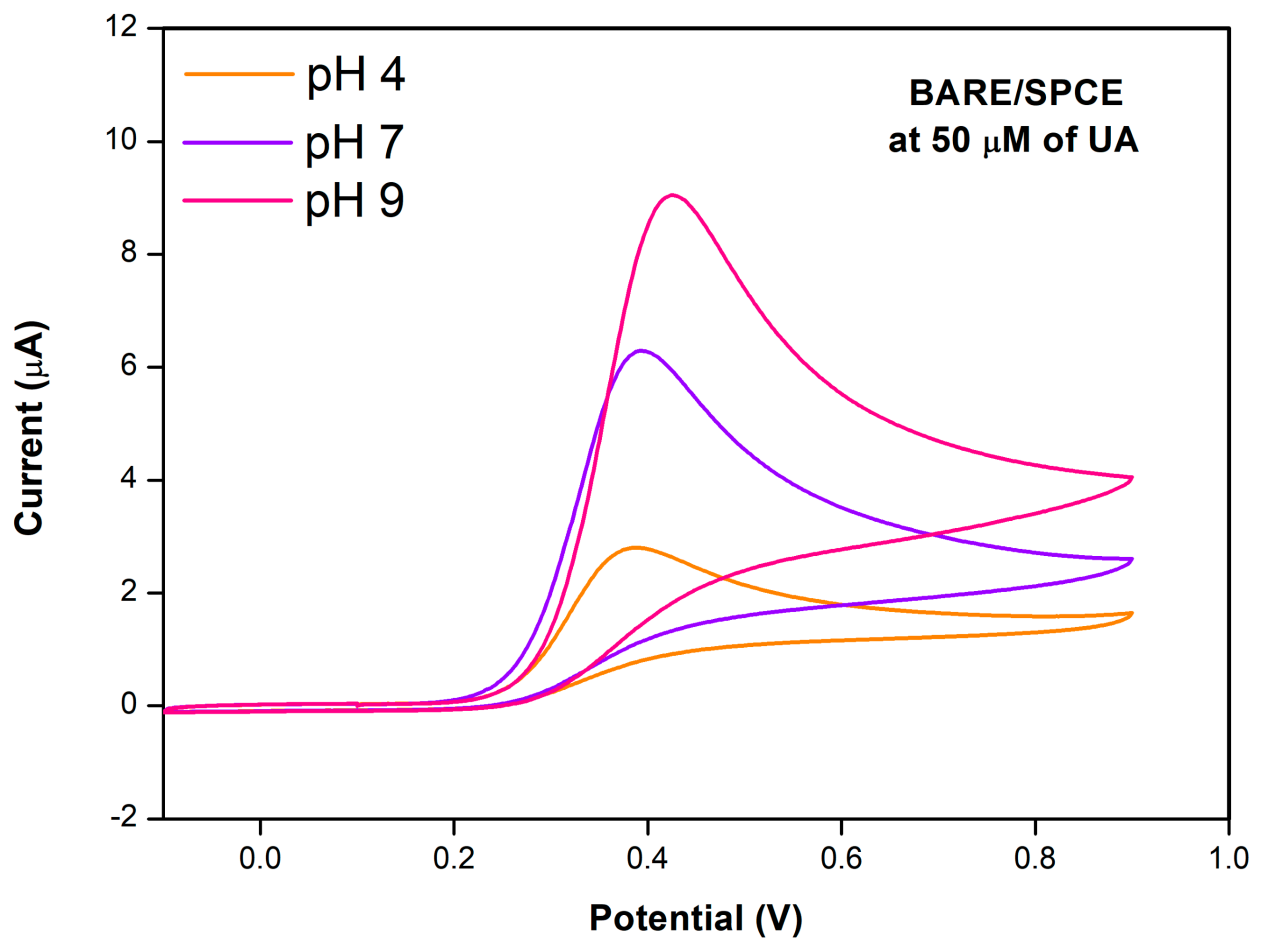
Cyclic voltammograms of the bare SPCE in the presence of 50 μM UA in PBS at different pH values.

In this section, the electroanalytical performance toward uric acid (UA) is evaluated using both unmodified and modified electrodes, namely bare SPCE, Co_3_O_4_/SPCE, and Ag_2_O/Co_3_O_4_ composites with different Ag_2_O loadings (Ag_2_O/Co_3_O_4_−0.5 and Ag_2_O/Co_3_O_4_−1.0). Since the pH of the solution plays a crucial role in determining the electroanalytical performance of the sensor [36,37] a pH optimization study was carried out by cyclic voltammetry at a scan rate of 0.05 V s^−1^ within a potential window of −0.1 to 0.9 V, in the presence of 50 μM UA using a bare SPCE in phosphate-buffered solution (PBS). Figure 7 shows the anodic current response as a function of pH. As observed, the current increased significantly with increasing pH, reaching its maximum value under basic conditions. This behavior indicates that alkaline PBS provides the most favorable environment for UA oxidation. Accordingly, pH 9 was selected as the optimal condition for subsequent electroanalytical measurements.

**Figure 8:**
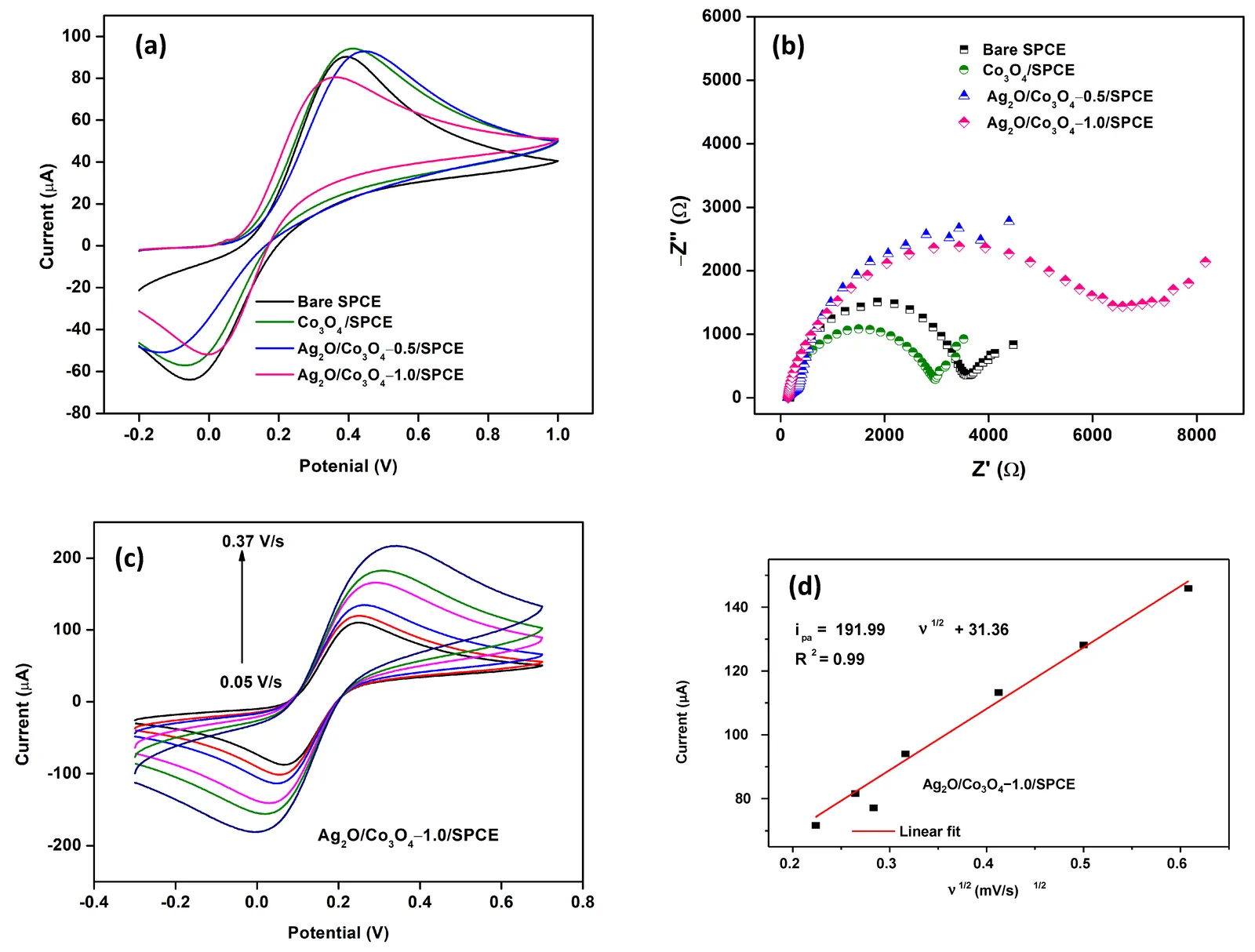
(**a**) CV tests, (**b**) EIS spectra with (inset) equivalent circuit of modified electrode in 10 mM ferrocyanide solutions, (**c**) study of scan rate on Ag_2_O/Co_3_O_4_−1.0/SPCE in ferrocyanide, and (**d**) linear response of anodic current peak vs. scan rate square root.

Cyclic voltammetry (CV) measurements were carried out in a 10 mM ferricyanide/ferrocyanide solution ([Fe(CN)_6_]^3−^/[Fe(CN)_6_]^4−^) at a scan rate of 0.05 V s^−1^ within the potential window of −0.2 to 1.0 V. Electrochemical impedance spectroscopy (EIS) measurements were also performed to investigate the interfacial charge-transfer properties of the electrodes. As shown in Figure 8a, the CV results indicate that Co_3_O_4_/SPCE and the Ag_2_O/Co_3_O_4_−0.5 composite exhibit enhanced electrochemical responses compared to the bare SPCE. In contrast, a significant decrease in the faradaic current is observed for the Ag_2_O/Co_3_O_4_−1.0 sample, likely due to the excessive Ag_2_O content, which may hinder charge transfer processes. The Nyquist plots reported in Figure 8b display the typical semicircular region at high frequencies followed by a linear tail at low frequencies. The fitting results (Table 3) show that the incorporation of Co_3_O_4_ significantly reduces the charge-transfer resistance (R_CT_) compared to bare SPCE, indicating improved electron-transfer kinetics. However, the addition of Ag_2_O leads to an increase in R_CT_, suggesting a higher resistance at the electrode/electrolyte interface, possibly due to altered interfacial properties and reduced charge mobility.

To gain deeper insight into the sensing mechanism, cyclic voltammetry (CV) measurements were performed at different scan rates in a 10 mM ferricyanide/ferrocyanide solution ([Fe(CN)_6_]^3−^/[Fe(CN)_6_]^4−^) (Figure 8c). As the scan rate increased from 0.05 to 0.37 V s^−1^, the anodic peak current progressively increased, accompanied by a slight shift toward higher potentials. A linear relationship between the anodic peak current and the square root of the scan rate is observed (Figure 8d), indicating that the electro-oxidation of ferrocyanide at the Ag_2_O/Co_3_O_4_−1.0/SPCE is governed by a diffusion-controlled process.

### 2.4. Electroanalytical Performance of Modified Electrodes

**Figure 9:**
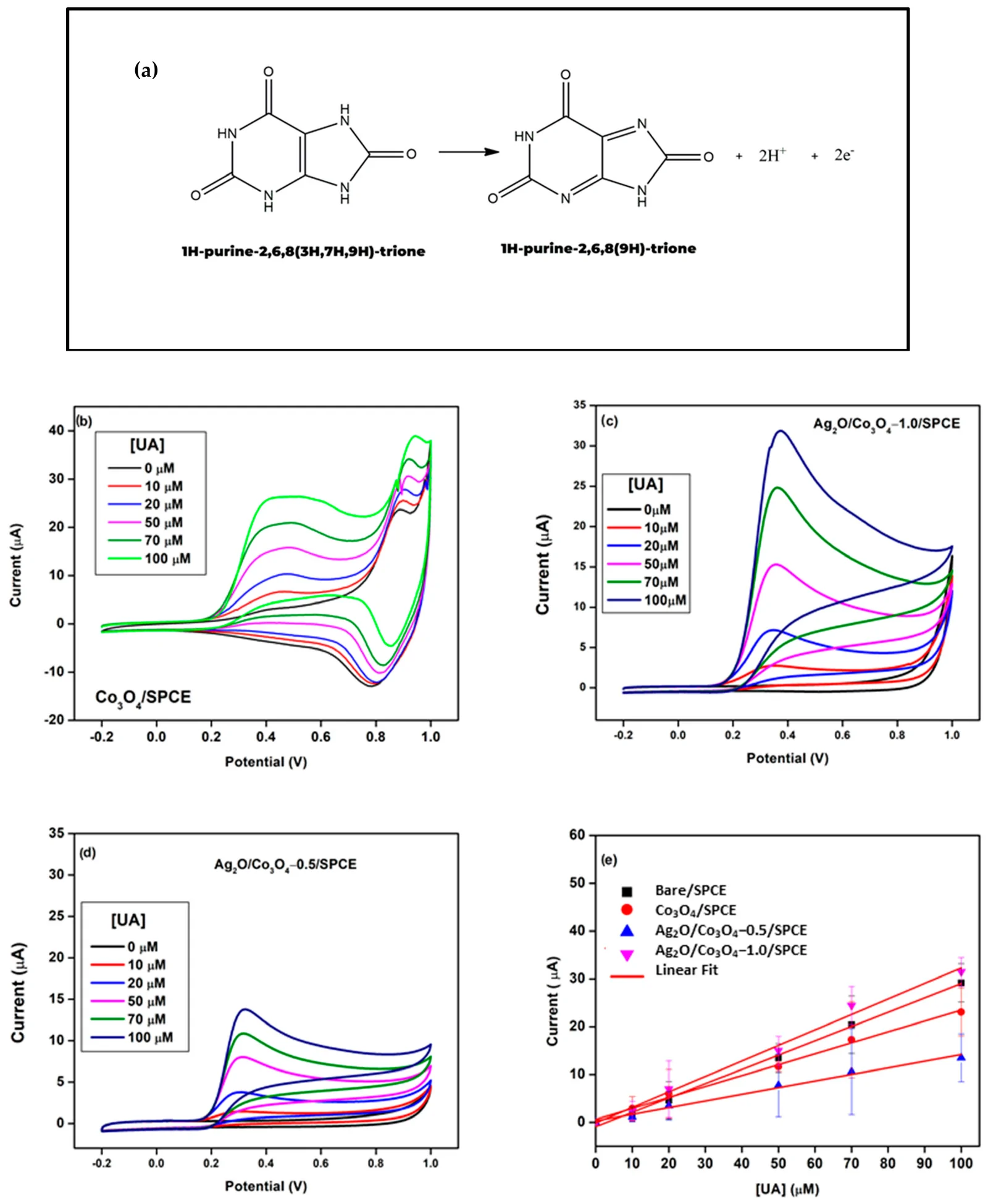
(**a**) Electro-oxidation reaction of uric acid (UA) at the electrode surface. Electroanalytical performance of (**b**) Co_3_O_4_/SPCE, (**c**) Ag_2_O/Co_3_O_4_−1.0/SPCE (**d**) Ag_2_O/Co_3_O_4_−0.5/SPCE toward UA determination, and (**e**) calibration curve. Data in the calibration curve are expressed as mean ± SD (n = 3), with error bars representing the standard deviation of three independent measurements.

Based on the results discussed above, cyclic voltammetry (CV) measurements were carried out to electroanalytically determine UA in the optimized phosphate-buffered solution (PBS, pH 9) at a scan rate of 0.05 V s^−1^ within the potential range of −0.2 to 1.0 V. The electrochemical reaction involved in UA oxidation is shown in Figure 9a [38]. The corresponding electroanalytical results are shown in Figure 9b–e. For the Co_3_O_4_/SPCE, two anodic peaks are observed (Figure 9b). The first peak, located at approximately 0.4 V, is attributed to the oxidation of uric acid to 1H-purine-2,6,8(9H)-trione, according to the reaction reported in Figure 9a. The second anodic peak can be assigned to the oxidation of Co_3_O_4_, although it appears shifted toward higher potential values compared with literature data, likely due to the alkaline PBS medium used in this study.

After the incorporation of Ag_2_O (Figure 9c,d), only the anodic peak associated with UA oxidation is clearly detected. Notably, the peak current increases for the Ag_2_O/Co_3_O_4_−1.0 modified electrode, suggesting enhanced electrocatalytic activity toward UA oxidation. Compared with Co_3_O_4_/SPCE, the disappearance of the Co_3_O_4_ oxidation peak may be attributed to improved kinetics of direct UA oxidation following Ag_2_O incorporation. Although the EIS results indicate an increase in R_CT_ upon Ag_2_O incorporation, this does not necessarily contradict the enhanced UA sensitivity. The ferrocyanide redox probe mainly reflects the overall interfacial charge-transfer resistance, whereas UA oxidation is strongly influenced by the number of catalytically active sites and the specific affinity of the electrode surface toward UA [37,39]. Thus, Ag_2_O incorporation may partially hinder electron transfer by increasing film resistance or blocking conductive pathways, while simultaneously enhancing UA oxidation through additional Ag-based catalytic sites [40]. Overall, these results suggest that Ag_2_O incorporation modifies the interfacial electrochemical environment of Co_3_O_4_ and promotes UA oxidation through additional catalytically active sites. This effect results in an enhanced anodic response despite the higher interfacial charge-transfer resistance observed with the ferrocyanide redox probe.

The calibration curves were obtained by plotting the anodic peak current as a function of UA concentration over the 0–100 μM range and fitting the experimental data using a linear regression model. For the Ag_2_O/Co_3_O_4_−1.0/SPCE, which exhibited the best electroanalytical performance, the calibration equation was *I_p_* (μA) = 0.3205 *C_UA_* (μM) − 0.1871, with a coefficient of determination R^2^ of 0.9920. Each concentration was measured in triplicate (n = 3), and the results are expressed as mean ± standard deviation (SD). The sensitivity, calculated by normalizing the slope of the calibration curve to the geometric electrode area, was 2.6 μA μM^−1^ cm^−2^, while the limit of detection (LoD) was 147 nM. The LoD was calculated according to LoD = 3.3σ/S, where σ represents the standard deviation of the analytical response and S is the slope of the calibration curve.

**Figure 10:**
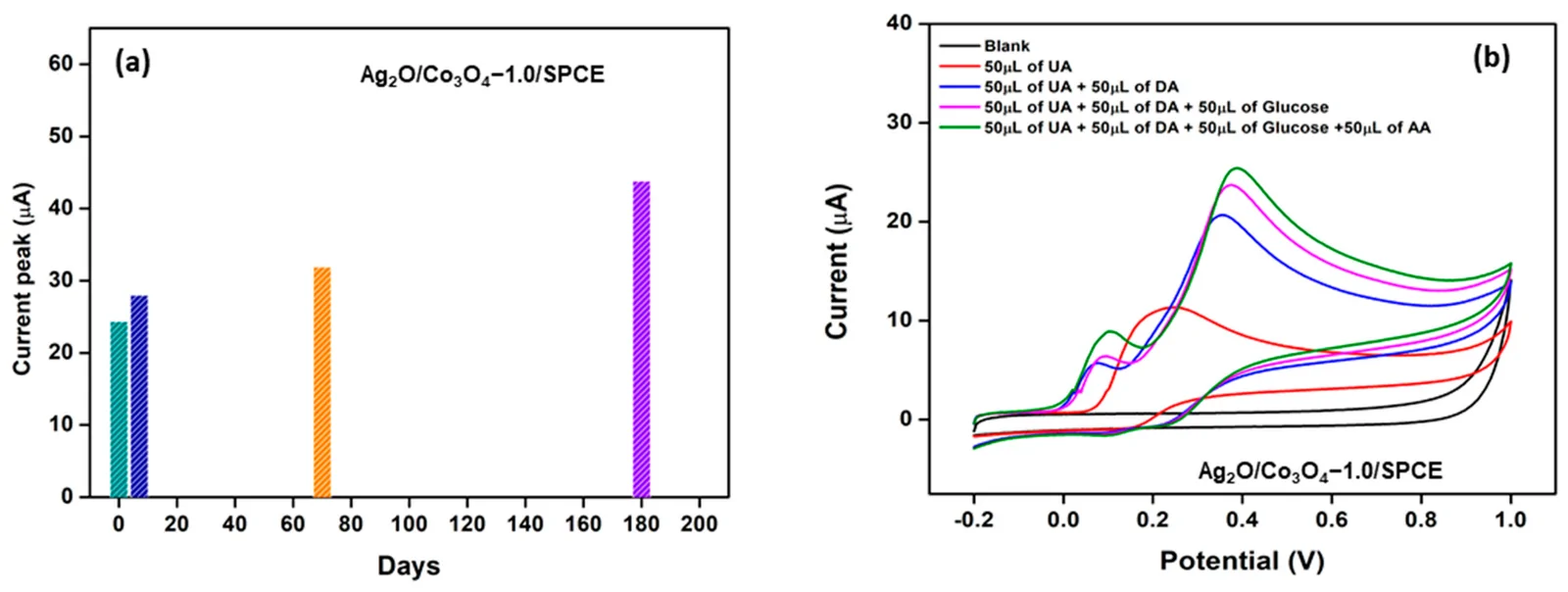
(**a**) Stability test after 5 (cyan bar), 10 (blue bar), 70 (orange bar), and 180 (violet bar) days of Ag_2_O/Co_3_O_4_−1.0/SPCE in PBS in the presence of UA at 70μM and (**b**) Selectivity test of Ag_2_O/Co_3_O_4_−1.0/SPCE in PBS in the presence of UA, DA, glucose, and ascorbic acid (AA).

Due to the remarkable electroanalytical performance of the Ag_2_O/Co_3_O_4_−1.0/SPCE, its stability was evaluated immediately after preparation and after 7, 70, and 180 days under the same experimental conditions used for the initial measurements (Figure 10a). The electrode exhibited stable electrochemical behavior throughout the evaluation period. A slight increase in the anodic peak current was observed after approximately 70 days, possibly reflecting minor changes in the electrode surface during storage. Nevertheless, the electrode maintained excellent long-term stability and reliable analytical performance for uric acid detection.

Since selectivity is a key parameter for sensor applications, the selectivity of the modified electrode was investigated in PBS containing 50 μM UA along with potential interfering species at comparable concentrations. As shown in Figure 10b, no oxidation peak is observed in the blank solution using the modified electrode. Upon the addition of UA, a well-defined anodic peak appears at approximately 0.2 V. In the presence of dopamine (DA), this peak shifts slightly toward lower potentials, while a second anodic peak corresponding to DA oxidation is observed at around 0.35 V. The simultaneous increase in both peak currents, along with minor potential shifts in the presence of glucose and ascorbic acid (AA), indicates that the electrode is capable of distinguishing between different analytes. These results demonstrate that the Ag_2_O/Co_3_O_4_−1.0/SPCE provides a selective electrochemical platform for the simultaneous and independent detection of UA and DA.

To further assess the practical applicability of the Ag_2_O/Co_3_O_4_−1.0/SPCE sensor, recovery experiments were performed in human saliva and human urine at three UA concentration levels (20, 50, and 80 µM). As summarized in Table 4, satisfactory recoveries were obtained in both matrices. In human saliva, recovery values ranged from 91 to 98%, whereas values between 96 and 99% were obtained in human urine. Notably, the slightly lower recovery observed at the lowest concentration in human saliva (91% at 20 µM) suggests a modest matrix-related effect at low analyte levels; however, the recovery improved to 98% at both 50 and 80 µM. In human urine, recoveries remained consistently close to 100% across the investigated concentration range. Furthermore, RSD values ranged from 1.98 to 6.11%, indicating satisfactory repeatability in the investigated biological matrices. Overall, these results support the applicability of the proposed sensor for UA determination in complex sample matrices and complement the selectivity and stability results obtained under controlled conditions.

Table 5 compares the electroanalytical performance of the proposed modified SPCE with previously reported systems in the literature, highlighting its competitive sensitivity and low limit of detection.

## 3. Experiment

### 3.1. Materials

Cobalt nitrate hexahydrate (Co(NO_3_)_2_·6H_2_O) and silver nitrate (AgNO_3_) (Sigma-Aldrich, Merck Life Science S.r.l., Milano Italy) were employed as cobalt and silver precursors, respectively. Deionized water was used as the solvent, while sodium hydroxide (NaOH) was used to adjust the pH. Hydrochloric acid (HCl) and nitric acid (HNO_3_) were used for the dissolution of the pure oxides. All chemicals and solvents were of analytical grade and were used without further purification.

### 3.2. Synthesis of Ag_2_O-Loaded Co_3_O_4_ Composites

Ag_2_O-loaded Co_3_O_4_ composites (hereafter referred to as Ag_2_O/Co_3_O_4_) were prepared by the sol–gel auto-combustion method. Ag_2_O and Co_3_O_4_ were first prepared as previously described [28]. Two composites with nominal Ag_2_O loadings of 0.5 and 1.0 wt% were prepared using 1.000 g of Co_3_O_4_ and 5.025 or 10.101 mg of Ag_2_O, respectively. The Ag_2_O loading was defined relative to the total mass of the composite as follows:

$$wt\%{\;Ag}_{2}O=\frac{m_{{Ag}_{2}O}}{m_{{Ag}_{2}O}+\;m_{{Co}_{3}O_{4}}}\;\times 100$$

**Figure 11:**
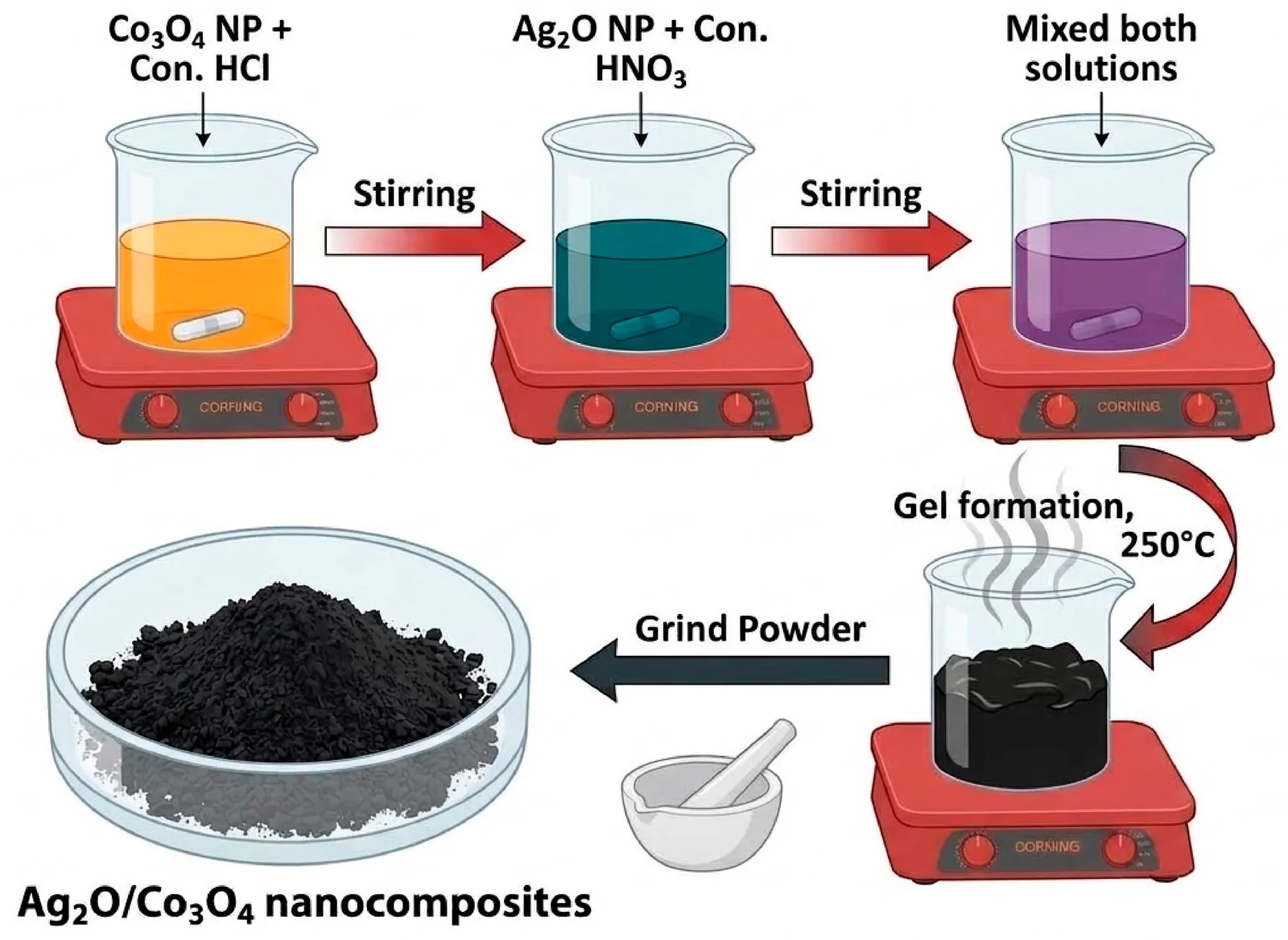
Schematic pathway of the Ag_2_O/Co_3_O_4_ synthesis.

The corresponding amounts of Co_3_O_4_ and Ag_2_O were dispersed in 10 mL of concentrated HNO_3_ and sonicated for 30 min. The resulting mixture was subsequently heated at 250 °C until gel formation due to solvent evaporation (Figure 11). Upon continued heating, the gel underwent auto-combustion, yielding the final Ag_2_O/Co_3_O_4_ composite powder. For clarity, the composites containing nominal Ag_2_O loadings of 0.5 and 1.0 wt% are hereafter denoted as Ag_2_O/Co_3_O_4_−0.5 and Ag_2_O/Co_3_O_4_−1.0, respectively.

### 3.3. Characterization

The morphology of the samples was analyzed using a scanning electron microscope (SEM, FEI QUANTA FEG 450, Thermo Fisher Scientific, Waltham, MA, USA) operated at 20 kV under high-vacuum conditions. The instrument was equipped with an energy-dispersive X-ray spectroscopy (EDX) detector (EDAX, Ametek, Tokyo, Japan) for elemental analysis. Attenuated total reflectance Fourier-transform infrared (ATR-FTIR) spectra were recorded using a Spectrum Two FT-IR spectrometer (PerkinElmer) equipped with a diamond attenuated total reflection (ATR) single-reflection accessory, with 30 scans accumulated for each spectrum. The specific surface area and porosity of the Ag_2_O/Co_3_O_4_ composites were determined by Brunauer–Emmett–Teller (BET) analysis based on nitrogen adsorption–desorption isotherms at 77 K, using a NOVA gas sorption analyzer (Quantachrome Instruments). Data acquisition and analysis were performed using NovaWin software (version 11.02). Before the measurements, the samples were dried under vacuum at 100 °C and degassed for 3 h.

### 3.4. Sensor Fabrication and Sensing Tests

#### 3.4.1. Gas Sensor Fabrication and Electrical Measurements

The Ag_2_O/Co_3_O_4_ materials were dispersed in deionized water to obtain a stable suspension. A 10 μL aliquot of the suspension was drop-cast onto the electrode surface on a ceramic substrate and allowed to dry at room temperature, forming the sensing layer. The fabricated sensor was tested in a sealed chamber equipped with a controlled gas-flow system. The electrical resistance was measured using a computer-controlled, signal-filtering multimeter (50 Hz ± 5%). During the gas-sensing measurements, controlled amounts of target gas were introduced into the chamber at room temperature. The sensor response (R) was defined as the ratio between the resistance in the presence of the target gas (R_(g)_) and that measured in air (R_(a)_), as expressed in Equation (6):

R = R_(g)_/R_(a)_ (6)

where R_(g)_ is the sensor resistance in the target gas atmosphere and R_(a)_ is the resistance in air.

#### 3.4.2. Screen-Printed Electrode Modification and Electrochemical Sensing

Co_3_O_4_, Ag_2_O, and Ag_2_O/Co_3_O_4_ composite powders were employed as electrode modifiers. Briefly, 3 mg of each powder was dispersed in 1 mL of isopropanol and ultrasonicated to obtain a homogeneous suspension. The screen-printed carbon electrode (SPCE, DropSens, 4 mm diameter) was modified via a spray-coating technique using an airbrush system equipped with a 7 cc gravity-feed cup and a 0.25 mm nozzle, operated at a pressure of 15–50 PSI. The suspension was uniformly sprayed onto the carbon working electrode (WE) surface, resulting in the formation of a thin and adherent film. The electrochemical experiments were carried out using a DropSens μStat 400 potentiostat/galvanostat equipped with DropView 8400 software for data acquisition and a Metrohm Autolab PGSTAT204 potentiostat/galvanostat (Metrohm Autolab B.V., Utrecht, The Netherlands) controlled using NOVA software (version 2.1) for electrochemical impedance spectroscopy (EIS) measurements. All electrochemical measurements used for the calibration study were performed in triplicate (n = 3). The results are reported as mean ± standard deviation (SD). Calibration curves were obtained by plotting the anodic peak current against UA concentration and fitting the experimental data using a linear regression model. The practical applicability of the Ag_2_O/Co_3_O_4_−1.0/SPCE sensor for UA determination was evaluated using human saliva and human urine as model matrices. Prior to electrochemical analysis, human saliva and urine samples were diluted 1:10 and 1:20, respectively, with PBS adjusted to pH 9. The diluted samples were spiked with UA to obtain final added concentrations of 20, 50, and 80 µM, selected within the linear calibration range of the proposed sensor. Electrochemical measurements were performed under the same experimental conditions used for UA calibration, and recovery values were calculated by comparing the experimentally determined UA concentration with the corresponding spiked concentration, while precision was expressed as relative standard deviation (RSD, %).

## 4. Conclusions

In conclusion, Ag_2_O/Co_3_O_4_ composites were successfully synthesized via a sol–gel auto-combustion method, yielding well-integrated binary oxide systems. Morphological, elemental, spectroscopic, and textural analyses supported the incorporation and homogeneous dispersion of the Ag-containing component within the Co_3_O_4_ matrix, together with modifications of the physicochemical properties of the resulting composites. Ag_2_O/Co_3_O_4_ composites exhibited enhanced hydrogen detection compared to pristine Co_3_O_4_, with the best performance achieved at lower Ag_2_O loading (0.5 wt%). The improvement is attributed to the synergistic interaction between Ag_2_O and Co_3_O_4_, which may promote catalytic activity and improve surface reactivity.

Ag_2_O/Co_3_O_4_ composites were also used to modify SPCEs, which demonstrated good performance toward uric acid detection in alkaline PBS (pH 9), achieving a sensitivity of 2.6 μA μM^−1^ cm^−2^ and a low limit of detection of 147 nM. The enhanced response is mainly attributed to the catalytic role of Ag_2_O and the increased number of active sites. Furthermore, the electrode exhibited good long-term stability and satisfactory selectivity toward common interfering species, although slight shifts in peak potential indicate possible interactions in multi-analyte systems. Overall, these findings demonstrate the versatility of the Ag_2_O/Co_3_O_4_ platform for distinct gas-sensing and electrochemical-sensing applications. The different optimal Ag_2_O loadings identified for H_2_ detection (0.5 wt%) and UA determination (1.0 wt%) indicate that the two sensing modalities are governed by different composition-dependent interfacial processes rather than by a single unified sensing mechanism. Accordingly, further studies correlating quantitative interfacial descriptors with both sensing responses will be required to establish a comprehensive structure–property–performance relationship.

## Figures and Tables

**Table 1:** Surface area, pore volume, and pore radius of pure Ag_2_O and Co_3_O_4_ and Ag_2_O/Co_3_O_4_ composites.

Samples	Surface Area (m^2^ g^−1^)	Pore Volume (cc g^−1^)	Pore Radius (Å)
Ag_2_O	78.12	0.109	21.679
Co_3_O_4_	59.63	0.141	17.165
Ag_2_O/Co_3_O_4_−0.5	30.75	0.039	16.316
Ag_2_O/Co_3_O_4_−1.0	38.44	0.041	16.442

**Table 2:** Comparison of the gas-sensing characteristics of selected Co_3_O_4_- and Ag_2_O-based sensors reported in the literature.

Sensor	Synthesis Method	Gas Detected	Temperature (°C)	Gas Concentration (ppm)	Ref
PANI/Ag_2_O/GO	Hydrothermal	NO_2_	100	25	[31]
SiO_2_-Co_3_O_4_	Sol–gel	H_2_, CO	300	10	[32]
Co_3_O_4_-Al-ZnO	Hydrothermal	NO_2_, CO	160,200	50–1000	[33]
Gr/Ag-Ag_2_O/Ppy	Polymerization	NH_3_	25	1000	[34]
Co_3_O_4_	Hydrothermal	CO	180	1–25	[35]
Ag_2_O/Co_3_O_4_–0.5	Sol–gel	H_2_	125	2000–10,000	This work

**Table 3:** Fitting results of different electrodes.

Electrode	Randles Circuit Parameters	
	R_s_ (Ω)	R_CT_ (KΩ)
Bare/SPCE	181	3.22
Co_3_O_4_/SPCE	136	2.66
Ag_2_O/Co_3_O_4_−0.5/SPCE	135	1.10
Ag_2_O/Co_3_O_4_−1.0/SPCE	128	3.97

**Table 4:** Recovery study for UA determination in human saliva and human urine using Ag_2_O/Co_3_O_4_−1.0/SPCE. Each recovery experiment was performed in triplicate (n = 3).

Real Sample	UA Added (µM)	Recovery (%)	RSD %
Human saliva	20	91	2.6
Human saliva	50	98	4.4
Human saliva	80	98	2.99
Human urine	20	96	5.7
Human urine	50	97	6.11
Human urine	80	99	1.98

**Table 5:** Comparative table between this work and other investigations on UA detection.

Sensor	Method	LoD	Sensitivity	Ref
FexOy/UNC/GCE	DPSV	0.29 μM	***	[41]
ZnO-Ag_2_O-Co_3_O_4_/GCE	I–V	89.14 pM	82.3323 μA μM^−1^ cm^−2^	[20]
r-GO/NPs	CV	3.6 µM	0.31 µA µM^−1^ cm^−2^	[36]
ZnO/rGO/GCE	CV	8.5 µM	0.00082 µA µM^−1^ cm^−2^	[42]
CuO	CV	0.6 µM	***	[43]
α-Fe_2_O_3_ NPs	DPV	2.5 nM	***	[44]
CoFe_2_O_4_-LSA/CT	AMP	0.3 µM	5140 µA µM^−1^ cm^−2^	[45]
Ag_2_O/Co_3_O_4_−1.0/SPCE	CV	147 nM	2.6 μA μM^−1^ cm^−2^	This work

***: not mentioned.

## Data Availability

All data supporting the findings of this study are included in the manuscript. Additional data are available from the corresponding author upon reasonable request.
